# Changes in Patterns of Social Role Combinations at Ages 25–26 among Those Growing Up in England between 1996 and 2015–16: Evidence from the 1970 British Cohort and Next Steps Studies

**DOI:** 10.1007/s10964-021-01477-1

**Published:** 2021-07-16

**Authors:** Thierry Gagné, Amanda Sacker, Ingrid Schoon

**Affiliations:** 1 International Centre for Lifecourse Studies in Society and Health (ICLS), London, UK; 2 Department of Epidemiology and Public Health, University College London, London, UK; 3 Department of Social Science, University College London, London, UK

**Keywords:** United Kingdom, Transition to adulthood, Social inequalities, Latent class analysis, 1970 British cohort study, Next Steps study

## Abstract

Changes across education, employment, and family life over the past 20 years challenges the capacity of previously established social role combinations to continue representing the experiences of young men and women born since the late 1980s. Latent class analysis was used to derive patterns of role combinations at ages 25–26 in those growing up in England, using data from 3191 men and 3921 women in the 1970 British Cohort Study (1996) and 3426 men and 4281 women in the Next Steps study born in 1989–90 (2015–16). Role combinations in 1996 were well defined by five patterns across genders: educated, work-oriented, traditional family, fragile family, and slow starters. Patterns in 2015–16 diverged across genders (e.g., disappearance of home ownership in the traditional family group among men and higher education as a group identifier among women) and included across genders fewer work-oriented, more slow starters, and a new group of “left behind” who are excluded from work and relationships. Young men and women born around 1990 experienced diverging role combinations characterized by increased delays and inequalities, with fewer being able to attain the milestones traditionally associated with the transition to adulthood by the mid-20s.

## Introduction

It has been argued that, since the 1970s, transitions into adulthood have become de-standardized, i.e., more diverse and protracted (Shanahan, 2000; Furlong & Cartmel, 2007). A worrying fact is that some transitions have become out of reach for many young people due to the “precarization” of employment and the unaffordability of housing since the early 2000s (Schoon & Bynner, 2017). These imply that the role configurations found in the 1980s, 1990s, and 2000s may characterize increasingly smaller groups and occur at more dispersed ages over time. In response, this study compares the experiences of two nationally representative age cohorts born 20 years apart in 1970 and 1989–90, coming of age in England in the 1990s and 2010s. The study adopts a person-centred approach to explore changes in the role combinations of men and women in their mid-20s, focusing on the prevalence of distinct role combinations and the potential for new, more precarious patterns emerging since the 1990s. It uses a gendered approach that considers men’s and women’s patterns distinctly, thereby shining new light on previous assessments of pathways to adulthood, and considers socio-economic inequalities through variations by family background (Ross et al., 2009; Schoon et al., 2012; Schoon & Lyons-Amos, 2016). This study focuses on people in their mid-20s based on the assumption that this age represents a meaningful point given that: 1) the majority of young adults has left education and made the step into the labor market by this age; 2) young adults continue to expect to have reached most of their adult milestones by this age (Schoon et al., 2012; Vespa, 2017; Billari et al., 2019). The study is based in Great Britain, where the median ages at which young adults started full-time employment, left their parents, and moved in with a partner were ages 19, 23, and 26 in 2017-18 (ONS 2019a, b, c).

### Identifying Patterns of Role Combinations

Young adulthood has been recognized as a distinct life phase between the end of adolescence and the start of fully-fledged adulthood for over thirty years now (Hogan & Astone, 1986; Buchmann, 1989; Shanahan, 2000; Settersten et al., 2005). Crucial milestones for young people in the third decade of life typically include: completing education, entering full-time paid employment, leaving the family household to live in one’s own residence, establishing a long-term relationship, and having children (Settersten et al., 2005). Some developmental theories have downplayed the importance of “objective” transition markers and highlighted the importance of subjective perceptions in identity formation (Arnett, 2000), sometimes neglecting the role of social change and rising inequalities in shaping these processes (Bynner, 2005; Côté & Bynner, 2008; Côté, 2014). Evidence however supports that reaching transition milestones and adhering with their related social norms continues to represent a critical dimension of identity development during the transition to adulthood (Shanahan et al., 2005; Benson & Elder, 2011; Eliason et al. 2015).

These role transitions are not independent of each other, and for a better understanding of transition experiences it is necessary to adopt a person-centred approach, examining how different roles weave together in young people’s lives (MacMillan & Copher, 2005). Guided by assumptions formulated within a life course approach to the study of human development (Shanahan, 2000; MacMillan & Eliason, 2003), research has identified distinct patterns in the pathways in which young people reach independent adulthood. In Great Britain, a study using data from two large cohorts born in 1958 and 1970 identified a five-fold typology to represent role combinations at age 26 in 1984 and 1996: 1) work-oriented homeowners without children, 2) highly educated renters without children, 3) parents in “traditional families” with jobs and mortgages, 4) parents in “fragile families” with poor employment and housing circumstances, and 5) slow starters yet to leave the parental home (Schoon et al., 2012). Whereas these patterns represented both cohorts, those aged 26 in 1996 were less likely to be in a traditional family and more likely to be educated without children. Men were more likely to be educated without children or slow starters and less likely to be in families. Supporting differences by social background, young adults were more likely to be educated without children and less likely to be in families if their parents had post-compulsory education, and more likely to be slow starters if they grew up in a two-parent home. Similar patterns were identified among people in their mid 20 s coming of age in the mid 1990s in the United States and Finland (Maggs et al., 2012; Salmela-Aro et al., 2012).

### New patterns of role combinations

Multiple changes question whether previous patterns of role combinations observed in young adults in their mid-20s still apply today. The first concerns the massive education expansion in the United Kingdom (UK) since the 1990s (Furlong & Cartmel, 2007), leading to the depreciation of secondary education and increased need for post-compulsory education. These changes are gendered, as women have been increasingly likely to participate in higher education and pursue a career instead of marriage and homemaking over time (ONS, 2019a). Following this shift, the past decades have witnessed the acceleration of the rise of non-stable forms of employment, particularly for those with fewer qualifications, and the decline of full-time employment as a guarantor of financial security, especially in young adults (Furlong, 2006; Schoon & Bynner, 2019). In the UK, increases in income among full-time employees has been 11 to 31% lower in those aged 18–29 compared with older age groups over the past two decades (Francis-Devine, 2020). The average house price, however, increased by over 200% during this period (HM Land Registry, 2020). Therefore, certain traditional pathways that were positively valued, such as being rapidly in full-time employment and a homeowner by the mid-20s, have likely decreased in prevalence as they have become harder to achieve and maintain (Schoon et al., 2012).

As a result of declines in financial opportunities, the second major change concerns the generalized delay in the transition out of parents’ household, with 37% more young adults living with parents at ages 25–26 in 2019 compared with the 1990’s in the UK (ONS, 2019b). Changes in education and work have been met with increases in the median ages at which young adults leave their parental home and have their first child (2 years later between 1997–2017) and at which they become homeowners (8 years later between 1997–2017) (ONS, 2019a). The poorer financial circumstances that followed the 2008 recession further exacerbated delays in family transitions (Studer et al., 2018). Transitions such as leaving the parental home and having children, which clearly delineated patterns identifying “slow starters” and those in traditional and fragile family pathways in the 1990s, may thus lead these role combinations to be less prevalent in the third decade of life since the start of this Millennium.

### New differences in role combinations by gender and social background

These changes in role combinations likely varied across subgroups. In keeping with the massive entry of women into the workforce and higher education, studies found that pathways became more similar between men and women towards the end of the 20th century (Winkler-Dworak & Toulemon, 2007; Ross et al., 2009). Few, however, have examined the extent to which this convergence included family life and housing transitions, and whether this has remained stable since the 1990s (Settersten et al., 2005; MacMillan & Copher, 2005; Amato et al., 2008; Oesterle et al., 2010; Schoon, 2015). Illustrating this in a US sample followed between the ages of 18 and 30, a study found two similar pathways for men and women yet key differences in a third “precarious” pathway, in which men were less likely to be working full-time whereas women were more likely to rapidly enter parenthood while unmarried (Oesterle et al., 2010). In keeping with the different social changes that young men and women have been facing over time (i.e., decline in job conditions and income for men, rising participation in higher education for women), transitions such as entering full-time employment and completing higher education may link to new gender differences at this age (Sironi, 2018).

Changes in role combinations may also include variation by family background. Young people from a less privileged background—in separated families where parents have few qualifications and routine jobs—have been more likely to enter employment and family formation at an earlier age compared with those from more privileged families (Schoon & Bynner, 2017). Despite increased participation in higher education across socioeconomic groups over the past two decades in the UK, inequalities have remained and led to new challenges, such as the selection of less privileged students into jobs that pay less and for which they are overqualified (UK Social Mobility Commission, 2019).

Parents may have been playing an increasing part in young adults’ capacity to successfully transition into adult roles over time (Ross et al., 2009; Schoon, 2015; Billari et al., 2019). There are multiple mechanisms through which parents influence this process: by shaping young adults’ expectations, their opportunities for positive transitions, and the capacity to navigate them successfully (Billari et al., 2019). That is, young adults in privileged families are expected to select themselves out of rapid transitions into work and family life, and use parental resources to complete a degree and start a career while not accumulating debt or delaying the move out of the parental home. In particular, entering a family-oriented pathway by the mid-20s may become increasingly likely among less privileged young adults in more recent decades (Mills et al., 2011, Schoon et al., 2012).

## Current Study

There is a lack of evidence on the transition patterns taken by more recent cohorts of young people, with previous findings being based on data collected before the massive education expansion, the precarization of employment and housing, and delays in independent living since the 1990s. These changes have affected men and women differently, with men experiencing more competition in the labor market and women further benefiting from the expansion of education. In response, this study assesses changes in role combinations among young men and women who grew up in England across two age cohorts born 20 years apart in 1970 and 1989–90. In contrast to previous studies which generally focused on single transitions such as employment or family formation, we use a person-based approach to examine changes across multiple interlinked transition milestones including education, employment, independent living (housing), and family formation. Specifically, latent class analysis is applied to identify patterns in role combinations at ages 25–26 in 1996 and 2015–16, and the distribution of these patterns by gender and parental socio-economic characteristics. First, following the assumption of increasing de-standardization, it is expected that transitions that have become more common (e.g., higher education participation) will have a weaker capacity to delineate patterns at this age. Second, following the precarization of some transitions it is assumed that less desirable patterns in terms of financial security have become more common. It is therefore expected that entry into these patterns continues to be predicted by parental characteristics, with the more precarious patterns being most strongly associated with social disadvantage. Finally, given that transitions have changed in different ways among men and women over time, it is expected that changes will be more pronounced in the most affected gender (e.g., employment in young men, education in young women).

## Methods

### Data

This study capitalizes on the similarity of data collected at ages 25–26 in two representative cohorts that grew up in England to capture transitions in generations born in 1970 and 1989–90: the 1970 British Cohort and the Next Steps studies.

The 1970 British Cohort study recruited 17,196 individuals born during a single week in April 1970 across Great Britain, followed them at ages 5, 10, 16, and 26, and still follow them today. The 1970 cohort was designed to study perinatal mortality and progressed to become multidisciplinary over time (Elliott & Shepherd, 2006; Chamberlain et al., 2013; University of London, 2016). At age 26, 12,290 cohort members were issued for fieldwork. Participants not contacted included those who had chosen to permanently withdraw from the study, been untraced for a lengthy period, emigrated out of the UK, or were deceased. At the end of the fieldwork period, 8332 cohort members followed since their birth participated, representing a follow-up rate of 68%.

The Next Steps study, formerly known as the Longitudinal Study of Young People in England, recruited 15,770 young people aged 13–14 in 2004, using annual follow-ups until ages 19–20, and another time at ages 25–26. The Next Steps study was developed to study educational attainment and the progression from compulsory education, and progressed to include the transition from education into work, relationships, and family life (University College London, 2018). At ages 25–26, 15,108 cohort members were issued for fieldwork. Participants not contacted included those who chose to permanently withdraw from the study, in prison, deceased, outside the UK, and identified to be in the armed forces or out of the survey for similar reasons. At the end of the fieldwork period, 3246 men and 4281 women participated, representing a follow-up rate of 51%.

Whereas the Next Steps cohort initially recruited participants in England, participants were invited in the ages 25–26 wave if they moved outside England as long as they stayed within the UK (0.7% lived in Wales, Scotland, or Northern Ireland at ages 25–26). To construct a comparable sample of young adults in the 1970 British Cohort Study, participants’ country of residence in adolescence was derived using data from the most recent time point across the ages 0–16 waves (i.e., if participants did not participate at age 16, information at age 10 was used, etc.). Only 3.1% of participants at age 26 did not have this information at ages 10 or 16. Using this definition, the analysis was done using data from the 3191 men and 3921 women in the 1970 cohort who were followed since birth, participated at age 26, and were identified to have lived in England growing up.

### Measures

#### Role transition indicators

The transition indicators included educational attainment, housing tenure, cohabitation with parents, economic activity, partnership status, and parenthood (Supplementary Table 1 presents descriptive statistics).

*Educational attainment* was measured using the National Vocational Qualifications (NVQ) scheme. Under this scheme, NVQ 1 and 2 represents different levels in secondary education, NVQ 3 represents pre-university training, NVQ 4 represents sub-degree programs (further education), and NVQ 5 represents university degrees (higher education). In the 1970 cohort, the items designed to measure educational attainment in the age 26 wave induced mis-responses, leading researchers to recommend against using this information (Dodgeon & Parsons, 2012). Using information collected at later waves, data on NVQ was derived by the authors for 91% of cohort members at age 26. In the Next Steps cohort, a NVQ variable cleaned by the Next Steps team was used to measure education at ages 25–26.

*Housing tenure* was measured in the 1970 and Next Steps cohorts asking “Do you own or rent your home or have some other arrangement?” and recoded into: 1) “main owner”, 2) “main renter”, or 3) “other” (living with parents or others rent-free, squatting, etc.). *Employment status* was measured using information derived by the 1970 and Next Steps teams and recoded into: 1) “full-time employed”, 2) “part-time employed”, 3) “unemployed”, 4) “full-time student”, 5) “at-home”, and 6) “other” (e.g., military, disability). *Cohabitation with parents* – 1) “living with at least one parent” or 2) “living without parents” – and *parenthood* – 1) “no children”, 2) “one child”, or 3) “two or more children” – were measured using household grid questionnaires asking about the number of household members and the nature of the participant’s relationship with them. Finally, *relationship status* was measured based on cohabitation and marital status and recoded into: 1) “single (not cohabiting)”, 2) “cohabiting with a partner”, 3) “cohabiting with a married partner”, and 4) “divorced, separated, or widowed”.

#### Social background indicators

Four characteristics were used to examine the distribution of patterns based on information at ages 0, 10, and 16 in the 1970 cohort and at baseline (ages 13–14) in the Next Steps cohort (Supplementary Tables 2 and 3 present descriptive statistics). *Mother’s age at birth* was measured using data at birth in the 1970 cohort, and data on participants’ birth date and the mother’s age at baseline in the Next Steps cohort (“Less than 20” / “20–24” / “25–29” / “30 or more”). *Parental education* was measured using the highest age among parents at which they left full-time education (“16 or less” / “17-18” / “19 or more”). *Parental social class* was measured using the highest level among parents with two different five-point schemes: 1) in the 1970 cohort, the Registrar General’s Social Class (RGSC, “I – Professional” to “V – Unskilled”); 2) in the Next Steps cohort, the National Statistics Socioeconomic Class (NS-SEC, “1 – Managerial & Professional” to “5 – Semi-routine & Routine”) (ONS, 2019a, b, c). For each scheme, a sixth residual category captures those whose parents were unemployed or out of the labor force (e.g., disabled, at-home). Whereas the RGSC and NS-SEC were both developed to capture social class, they are not directly comparable as the RGSC combined occupations based on skill whereas the NS-SEC combines them based on employment relationships and conditions (ONS, 2019a, b, c). *Family structure* was measured using data on household composition at age 16 (using data at age 10 if it was missing at age 16) in the 1970 cohort and at baseline in the Next Steps cohort (Living with both parents / Not). Other covariates included participants’ region of residence in adolescence, using the official region definitions available at the time, and race/ethnicity.

### Statistical Analysis

Social role combinations at ages 25–26 were first modeled using latent class analysis (LCA) in the four groups defined by gender and cohort separately. To derive the appropriate number of groups, solutions were compared with an increasing number of classes. Following best practices, four fit values were used to evaluate the best-fitting solution: 1) standard and sample-size-adjusted Bayesian Information Criteria (BIC) (lower value indicates a parsimonious solution), 2) Vuong-Lo-Mendell-Rubin test (non-significant *p* value indicates that a model with *k* classes is not an improvement over a model with *k* - 1 classes), 3) entropy (higher value indicates that individuals are well discriminated when classified into their most likely class), 4) the size of the smallest class when participants are classified into their most likely class (classes with a very small size being unlikely to be substantive) (Nylund et al., 2007; Ross et al., 2009; Asparouhov & Muthén, 2012; Green, 2014).

The distribution of patterns in the 1970 and Next Steps cohorts was then examined by regressing participants’ most likely class on the four parental variables and covariates using multinomial logistic regression models. To better interpret associations, adjusted marginal probabilities of being in a role combination group were examined across predictor categories based on fully-adjusted models. Simulation studies have shown that regressing the most likely class was appropriate to predict class membership, when entropy values and sample sizes were adequate, with the combined use of a lower statistical significance threshold (Clark & Muthén, 2009). Therefore, a threshold of *p* < 0.01 (and 99% confidence intervals) was used to interpret findings as statistically significant.

Analyses were weighted for non-response using the longitudinal weight developed by the Next Steps team and a similar weight for the 1970 British Cohort created by the authors using nine birth variables: ages at which the mother and father left education, employment status of the mother and father, father’s social class (mother’s if the data on the father was missing), mother’s marital status, sex, and birth weight. Analyses in the Next Steps dataset included the design weight variables to account for its complex sampling at baseline. Latent class analyses were performed with data on all participants, using full information maximum likelihood for missingness, in MPlus 7 (Muthén & Muthén, 1998). Regressions were performed with data on all participants, in 20 imputed datasets using multiple imputation by chained equations for missingness, in Stata 16 (Royston & White, 2011, StataCorp, 2019).

## Results

### Transition Characteristics at Ages 25–26 in 1996 and 2015–16

Each cohort’s characteristics are described using proportions weighted for non-response and attrition in Table 1, and frequencies with missing cases in Supplementary Tables 1 and 2.

**Table 1:** Sample characteristics in the 1970 British cohort (1996) and next steps (2015–16) studies.

Variable	1970 BCS *N* = 7112		Next Steps *N* = 7707	
	Men *N* = 3191	Women *N* = 3921	Men *N* = 3246	Women *N* = 4281
	W%	W%	W%	W%
Education (NVQ)				
No qualifications	12.2	12.8	9.3	8.6
NVQ 1: CSE/GCSE−	7.8	9.4	17.8	13.3
NVQ 2: 0-level/GCSE+	29.9	33.2	26.2	23.9
NVQ 3: A-level	16.0	12.2	15.4	17.3
NVQ 4: Higher qual	29.4	27.5	19.1	24.5
NVQ 5: Degree	4.8	5.1	12.2	12.4
Housing tenure				
Main owner	38.6	46.7	19.4	21.5
Rent	28.3	30.4	41.4	49.1
Rent-free & other	33.0	22.9	39.2	29.4
Living with parents				
Living with parents	27.9	19.2	35.3	26.4
Living without parents	72.1	80.8	64.7	73.6
Economic activity				
FT employed	83.5	63.8	75.0	57.9
PT employed	2.6	13.0	7.2	17.2
Unemployed	7.4	2.8	9.1	6.3
FT student	3.0	2.1	4.1	5.1
At-home	0.4	14.9	0.7	10.8
Other	3.0	3.6	3.8	2.7
Relationship status				
Single	49.8	34.4	64.5	53.9
In couple	24.3	25.4	27.5	32.1
Married	22.4	35.3	7.7	12.9
Div./Sep./Widowed	1.9	4.9	0.3	1.1
Living with children				
No children	82.8	68.8	84.0	67.3
One child	11.4	17.2	9.2	16.3
Two or more children	5.7	14.0	6.7	16.4

W% = Weighted for non-response and attrition.

*NVQ* national vocational qualifications, *FT* full-time, *PT* part-time, *Div.* divorced, *Sep.* separated.

At the age of 26 in 1996, 12% of men and 13% of women had no qualifications (NVQ 0) and 50% of men and 46% of women had completed post-secondary education (NVQ levels 3–5). Thirty-nine percent of men and 47% of women were homeowners. Women were less likely to be living with their parents (19 vs 28%), and more likely to be married (35 vs 22%) and have one (17 vs 11%) or multiple (14 vs 6%) children compared to men. Women were also less likely to be employed full-time (64 vs 84%) and unemployed (3 vs 7%), and more likely to be employed part-time (13 vs 3%) and stay at home (15 vs <1%) compared to men.

At the ages of 25–26 in 2015–16, 9% of men and women had no qualifications (NVQ 0) and 47% of men and 54% of women had completed post-secondary education (NVQ levels 3–5). Women were more likely to have completed A-levels (NVQ 3) (17 vs 15%) and higher qualifications (NVQ 4) (25 vs 19%) compared to men. Nineteen percent of men and 21% of women were homeowners. Women were more likely to be living without parents (74 vs 65%), renting their own place (49 vs 41%), cohabiting with a partner (32 vs 28%) or being married (13 vs 8%), and more likely to have one (16 vs 9%) or multiple (16 vs 7%) children in their household compared to men. Women were also less likely to be employed full-time (58 vs 75%) or unemployed (6 vs 9%), and more likely to be employed part-time (17 vs 7%) or stay at home (11 vs <1%) compared to men.

Comparing cohorts, the number of young adults with university degrees (NVQ 5) increased but home ownership decreased by 50% among men and 54% among women between cohorts. Young men and women in the Next Steps cohort were 27 and 38% more likely to be living with parents and 66 and 63% less likely to be married compared to the 1970 cohort. Young men and women in the Next Steps cohort were 10 and 9% less likely to work full-time, 177 and 32% more likely to work part-time, and 23 and 125% more likely to be unemployed compared to the 1970 cohort. The proportions of young adults with children did not significantly vary between cohorts.

### Role Combinations at Ages 25–26 in 1996 and 2015–16

Tables 2 and 3 report the best-fitting solutions for men and women in the 1970 and Next Steps cohorts (Supplementary Tables 4–5 present LCA results). Entropy values suggested that participants were well represented by their most likely class in the 1970 cohort (0.87 in men and 0.85 in women), and adequately represented by their most likely class in the Next Steps cohort (0.72 in men and 0.75 in women).

**Table 2:** Role combinations at age 26 in those who grew up in England. 1970 British Cohort study (1996).

	Men *n* = 3191					Women *n* = 3921				
Class	1	2	3	4	5	1	2	3	4	5
Name	FF	SS	TF	E	W	FF	SS	TF	E	W
Prevalence (%)	3.1	31.0	14.7	19.9	31.3	12.4	18.6	16.5	16.4	36.1
	%	%	%	%	%	%	%	%	%	%
Education										
No qualifications	**46.4**	10.4	13.9	10.5	9.9	**35.0**	10.8	14.3	7.5	8.0
NVQ 1: CSE	10.1	8.6	10.0	7.5	6.1	11.6	10.0	14.4	5.6	7.4
NVQ 2: 0-level	22.5	32.4	**46.7**	17.9	27.4	38.5	32.4	**48.4**	16.7	31.0
NVQ 3: A-level	12.6	16.2	13.7	10.2	20.0	6.2	12.8	1.5	8.0	15.6
NVQ 4: Higher	7.4	28.8	14.2	**40.7**	33.2	7.5	29.0	11.1	**48.1**	32.9
NVQ 5: Degree	0.9	3.6	1.4	**13.2**	3.4	1.2	4.9	0.2	**14.2**	5.1
Housing tenure										
Main owner	0.0	2.6	**64.4**	2.2	**85.6**	7.3	3.0	**76.3**	1.5	**85.4**
Rent	73.7	5.8	29.2	**87.8**	9.3	**89.4**	3.1	16.4	**85.8**	9.3
Rent-free/other	26.3	**91.6**	6.4	10.0	5.1	3.3	**93.9**	7.3	12.7	5.3
Living with parents										
Living without	**85.6**	11.1	**97.0**	**100.0**	**98.8**	**96.7**	**3.8**	**95.5**	**100.0**	**99.4**
Living with parents	14.4	**88.9**	3.0	0.0	1.2	**3.3**	**96.2**	4.5	0.0	0.6
Economic activity										
Full-time employed	32.1	**79.7**	**96.2**	67.7	**97.2**	6.0	**80.5**	15.8	**68.8**	**93.4**
Part-time employed	7.9	2.8	0.5	5.5	0.9	**20.4**	7.0	**39.3**	7.8	3.7
Unemployed	**41.9**	10.2	1.8	11.1	0.9	3.5	2.9	1.6	8.0	0.9
Full-time student	0.0	3.3	0.6	10.0	0.2	0.4	3.3	0.1	8.0	0.5
At-home	7.8	0.0	0.3	0.0	0.1	**67.7**	1.4	**38.8**	0.0	0.0
Other	10.3	4.0	0.6	5.7	0.7	1.9	4.9	4.4	7.4	1.6
Relationship status										
Single	17.1	94.5	0.0	70.2	23.6	29.2	**92.0**	0.0	58.6	13.8
In couple	**46.3**	2.4	26.9	25.6	**39.5**	31.1	2.2	16.3	34.7	35.7
Married	**31.5**	1.2	**72.7**	2.7	**34.6**	26.1	1.6	**78.0**	2.6	**48.2**
Div./Sep./Widowed	5.1	1.9	0.4	1.6	2.3	13.6	4.2	5.7	4.1	2.4
Living with children										
No children	33.1	99.9	0.0	**100.0**	**100.0**	2.0	96.2	7.3	97.1	94.2
One child	**34.1**	0.0	**69.8**	0.0	0.0	**44.4**	3.8	**49.3**	2.9	5.8
Two or more children	**32.8**	0.1	**30.2**	0.0	0.0	**53.6**	0.0	**43.4**	0.0	0.0

Estimates represent the proportion of participants with the characteristic in a latent class. Estimates on the top of columns represent the proportion of participants in each class when classified according to their most likely class. Estimates were bolded to help the interpretability of classes.

Pathways: *FF* fragile family, *SS* slow starter, *TF* traditional family, *E* educated, *W* work-oriented.

**Table 3:** Role combinations at ages 25–26 in those who grew up in England.

	Men *n* = 3426					Women *n* = 4281					
Class	1	2	3	4	5	1	2	3	4	5	6
Name	LB	SS	TF	E	W	FF	LB	SS	TF	E	W
Prevalence (%)	16.8	37.1	10.5	18.4	17.1	14.4	11.3	29.7	9.6	19.4	15.7
	%	%	%	%	%	%	%	%	%	%	%
Education											
No qualifications	**31.3**	2.8	**18.2**	0.0	6.3	**21.6**	**19.6**	5.8	5.5	2.8	2.3
NVQ 1: GCSE−	**44.8**	9.3	**47.2**	0.8	9.6	**30.2**	**37.3**	7.8	15.4	2.0	1.5
NVQ 2: GCSE+	17.5	30.2	28.6	13.6	38.1	**27.6**	**27.8**	20.2	**43.7**	17.5	19.0
NVQ 3: A-level	0.3	21.6	4.2	24.7	13.8	7.5	7.5	19.3	17.3	23.7	23.1
NVQ 4: Higher	6.2	21.5	1.6	**31.1**	24.4	12.8	5.7	**28.6**	13.7	**32.4**	**39.1**
NVQ 5: Degree	0.0	14.6	0.1	**29.8**	7.7	0.3	2.1	**18.3**	4.5	**21.7**	**15.1**
Housing tenure											
Main owner	13.8	10.9	0.0	14.1	**61.9**	0.4	5.1	12.0	**54.2**	0.0	**65.6**
Rent	43.7	13.8	**96.7**	**76.7**	29.6	**99.3**	**62.2**	13.5	38.8	**93.5**	27.6
Rent-free/other	42.5	**75.2**	3.3	9.2	8.6	0.3	32.6	**74.6**	7.0	6.5	6.8
Living with parents											
Living without	50.0	50.0	**99.0**	**100.0**	**97.1**	**100.0**	64.1	30.3	**95.7**	**96.3**	**100.0**
Living with parents	50.0	50.0	1.0	0.0	2.9	0.0	35.9	**69.7**	4.3	3.7	0.0
Economic activity											
Full-time employed	44.9	74.9	74.6	**82.9**	**97.6**	7.4	8.7	**77.5**	28.0	**84.7**	**91.1**
Part-time employed	11.6	9.8	4.9	3.6	2.4	**26.5**	**40.5**	9.7	**38.3**	6.0	4.4
Unemployed	**25.4**	8.5	11.0	2.7	0.0	10.7	**21.6**	5.4	1.9	0.9	1.1
Full-time student	0.7	5.4	0.2	10.8	0.0	6.7	2.9	6.3	0.0	8.4	3.1
At-home	1.2	0.2	4.1	0.0	0.0	**46.9**	11.4	0.0	**29.4**	0.0	0.0
Other	**16.2**	1.3	5.2	0.0	0.0	1.8	14.9	1.0	2.4	0.0	0.3
Relationship status											
Single	**97.4**	**92.5**	4.2	**65.9**	2.8	43.7	**74.4**	**91.5**	1.4	**57.4**	10.8
In couple	2.2	5.1	**79.4**	32.8	**66.3**	**40.6**	14.6	7.0	**49.3**	**42.5**	**61.1**
Married	0.0	2.0	15.8	1.4	**30.7**	13.9	7.6	0.8	**48.4**	0.0	**27.7**
Div./Sep./Widowed	0.4	0.4	0.6	0.0	0.2	1.9	3.4	0.7	1.0	0.1	0.5
Living with children											
No children	**93.7**	**97.0**	17.4	**98.2**	70.1	0.0	47.9	**98.7**	4.7	**94.5**	**91.5**
One child	6.3	2.3	**33.3**	1.6	21.4	**27.4**	**43.1**	0.8	**45.1**	5.5	8.2
Two or more children	0.0	0.6	**49.3**	0.2	8.4	**72.6**	9.0	0.5	**50.2**	0.0	0.4

Next Steps study (2015–16).

Estimates represent the proportion of participants with the characteristic in a latent class. Estimates on the top of columns represent the proportions of participants in each class when they are classified according to their most likely class. Estimates were bolded to help the interpretability of classes.

Pathways: *FF* fragile family, *SS* slow starter, *TF* traditional family, *E* educated, *W* work-oriented, *LB* left behind.

The findings for those growing up in England replicated the findings previously found for Britain (Schoon et al., 2012). For men (M) and women (W), five groups were found: 1) slow starters (M: 31% and W: 19%), 2) fragile families (M: 3% and W: 12%), 3) traditional family (M: 15% and W: 17%), 4) educated (M: 20% and W: 16%), and 5) work-oriented (M: 31% and W: 36%). The nature of classes was similar between men and women, with one difference for economic activity in family pathways: men were more likely to report full-time employment or unemployment, whereas women were more likely to report part-time employment or being at-home.

Among men in 2015–16, four of the five groups identified in 1996 were found: 1) educated (18%), 2) traditional family (11%), 3) slow starter (37%), and 4) work-oriented (17%). The traditional family group was no longer associated with home ownership (from 64 to 0%) and marriage (from 73 to 16%) compared with 1996. Replacing the fragile family group found in the 1970 cohort, a new “left behind” group (17%) characterized by a lack of qualifications and a high risk of unemployment was found. The label “fragile family” was no longer applicable for men in 2015–16 as those in the “left behind” group included mostly single men without children.

Among women in 2015–16, all five groups identified in the 1970 cohort were found: 1) educated (19%), 2) traditional family (10%), 3) fragile family (14%), 4) slow starters (30%), and 5) work-oriented (16%). A new sixth “left behind” group (11%) of women likely to have no qualifications, full-time employment, or relationships was also found. Despite the high probability of being single in this group, among women this group had a notable likelihood of having children (52%), which may allude to the presence of early and single mothers in this group. Supporting this, the mean age of the oldest child was higher in this group (4 years old) compared to the traditional family and fragile family (2 years old each) groups.

Table 4 presents a summary of patterns across cohorts. The “slow starter” group increased from 31 to 38% in men and from 19 to 30% in women. The “fragile family” group remained similar in size (12–14%) in women, but was no longer found in men. The new “left behind” group comprised 17% of men and 11% of women in 2015–16. Other patterns varied in meaning across cohorts according to educational attainment, housing tenure, and marital status. The “educated” group, no longer different in terms of educational attainment from “slow starter” and “work-oriented” groups among women, was similar in size (16–20%) across cohorts. The “work-oriented” group, defined in part by home ownership, decreased from 31 to 17% in men and from 36 to 16% in women. Finally, the “traditional family” group (where home ownership and marriage was largely absent among men in 2015–16) decreased from 15 to 11% in men, and from 17 to 10% in women.

**Table 4:** Summary of role combinations at ages 25–26 in those who grew up in England.

Men				Women			
1996		2015–16		1996		2015–16	
%	Description	%	Description	%	Description	%	Description
3	Fragile family			12	Fragile family	14	Fragile family
	Low qualifications				Low qualifications		Least qualifications
	Without parents, renting				Without parents, renting		Without parents, renting
	Not full-time, unemployed				Part-time or at-home		Part-time or at-home
	In relationship				In relationship		In relationship
	With children				With children		With children
		17	Left behind			11	Left behind
			Least qualifications				Least qualifications
			Parents or independent living				Parents or new arrangement
			Not full-time, unemployed				Part-time or unemployed
			Single				Single
			No children				May include children
31	Slow starter	37	Slow starter	19	Slow starter	30	Slow starter
	Post-mandatory qual.		Post-mandatory qual.		Post-mandatory qual.		Post-mandatory qual.
	With parents		Parents or new arrangement		With parents		With parents
	Working full-time		Working full-time		Working full-time		Working full-time
	Single		Single		Single		Single
	No children		No children		No children		No children
15	Traditional family	11	Traditional family	17	Traditional family	10	Traditional family
	Mandatory qualifications		Mandatory qualifications		Mandatory qualifications		Mandatory qualifications
	Without parents, owning		Without parents, renting		Without parents, owning		Without parents, owning
	Working full-time		Working full-time		Part-time or at-home		Part-time or at-home
	Married		In couple		Married		In couple
	With children		With children		With children		With children
20	Educated	18	Educated	16	Educated	19	Educated
	Highest qualifications		Highest qualifications		Highest qualifications		Post-mandatory qual.
	Without parents, renting		Without parents, renting		Without parents, renting		Without parents, renting
	Full-time (or studying)		Full-time (or studying)		Full-time (or studying)		Full-time (or studying)
	Mostly single		Mostly single		Mostly single		Mostly single
	No children		No children		No children		No children
31	Work-oriented	17	Work-oriented	36	Work-oriented	16	Work-oriented
	Post-mandatory qual.		Post-mandatory qual.		Post-mandatory qual.		Post-mandatory qual.
	Without parents, owning		Without parents, owning		Without parents, owning		Without parents, owning
	Working full-time		Working full-time		Working full-time		Working full-time
	In relationship		In relationship		In relationship		In relationship
	No children		No children		No children		No children

*Qual.* qualifications.

### Differences in Role Combinations at Ages 25–26 in 1996 and 2015–16

Tables 5 and 6 present the distribution of patterns at ages 25–26 in the 1970 and Next Steps cohorts across parental indicators, reporting the adjusted marginal probabilities of being in a group across categories. Estimates should be interpreted as the “direct” effect of predictors on group membership (e.g., the “total” effect of parental education may be explained indirectly by the adjustment for parental social class).

**Table 5:** Inequalities in role combination patterns at ages 25–26 in those growing up in England.

	Men (*n* = 3191)					Women (*n* = 3921)				
Name	FF	SS	TF	E	W	FF	SS	TF	E	W
Prevalence (%)	3.1	31.0	14.7	19.9	31.3	12.4	18.6	16.5	16.4	36.1
Predictors	%	%	%	%	%	%	%	%	%	%
Mother’s age at birth										
<20	5.2	24.4	17.4	20.4	32.5	16.3	12.5	22.4	16.7	32.2
20–24 (ref.)	3.3	27.7	17.6	17.7	33.7	14.0	16.6	16.6	14.9	37.8
25–29	2.4	30.9	13.7	21.3	31.7	**9.7**	20.4	15.8	16.0	38.0
30 or more	2.6	**39.3**	**10.0**	21.2	**27.1**	11.7	**21.5**	15.1	**19.1**	32.7
Parents’ education										
Left FT education at ages ≤ 16	3.3	31.9	**16.7**	**17.1**	31.1	13.4	18.8	**18.1**	**13.2**	36.5
Left FT education at ages 17–18	2.4	31.9	8.4	**22.7**	34.5	9.0	17.8	13.8	21.2	38.2
Left FT education at ages ≥ 19 (ref.)	2.3	26.7	9.2	30.9	30.8	9.4	20.5	9.7	25.8	34.6
Parental social class (RGSC)										
I – Professional (ref.)	0.4	23.7	7.6	29.4	38.9	3.2	17.4	10.4	24.3	44.7
II – Managerial and technical	1.3	29.9	**13.7**	**21.5**	33.5	**9.2**	18.2	14.6	18.1	39.9
III – Skilled	**3.8**	**32.6**	**15.2**	**17.2**	31.2	**14.1**	19.6	**17.4**	**14.4**	**34.5**
IV – Partly-skilled	**5.3**	**38.0**	**17.0**	**17.5**	**21.4**	**19.4**	19.3	**22.1**	**11.0**	**28.2**
V – Unskilled	6.7	30.9	25.3	18.5	**18.6**	**22.4**	15.9	18.1	16.1	27.6
Not applicable	3.0	29.1	17.0	26.8	24.1	16.1	12.4	31.8	13.0	26.7
Family structure										
Living with two parents (ref.)	2.9	32.1	14.2	18.8	31.9	11.3	19.1	16.3	16.0	37.3
Living without two parents	3.6	27.0	16.2	**24.2**	28.9	**16.4**	16.6	17.4	18.2	**31.4**

1970 British Cohort study (1996).

Estimates are adjusted marginal probabilities from a multinomial logistic regression model, weighted for non-response in 20 imputed datasets. Predictors were entered in a single model with region of residence in adolescence and ethnic group (White/Other). Bolded estimates differ from the reference category at the *p* < 0.01 level.

*RGSC* registrar general’s social class. Pathways: *FF* fragile family, *SS* slow starter, *TF* traditional family, *E* educated, *W* work-oriented.

**Table 6:** Inequalities in role combination patterns at ages 25-26 in those growing up in England

	Men (*n* = 3426)					Women (*n* = 4281)					
Name	LB	SS	TF	E	W	FF	LB	SS	TF	E	W
Prevalence (%)	16.8	37.1	10.5	18.4	17.1	14.4	11.3	29.7	9.6	19.4	15.7
Predictors	%	%	%	%	%	%	%	%	%	%	%
Mother’s age at birth											
<20	15.4	25.4	21.2	18.5	19.5	24.9	12.4	22.4	15.9	14.6	9.8
20–24 (ref.)	20.2	36.0	11.8	14.9	17.2	18.0	12.2	26.9	12.6	16.6	13.6
25–29	15.0	38.4	9.8	19.0	17.8	**11.2**	11.9	30.7	8.8	19.8	17.6
30 or more	16.7	40.1	**6.3**	**20.6**	16.3	**10.7**	9.7	**33.5**	**6.8**	**22.5**	16.7
Parents’ education											
Left FT education at ages ≤ 16	**21.1**	36.1	**12.5**	**12.5**	17.9	16.0	**13.7**	30.8	8.8	**14.5**	16.1
Left FT education at ages 17–18	15.3	39.5	10.5	**15.7**	18.9	13.3	10.1	30.1	10.9	**20.0**	15.7
Left FT education at ages ≥ 19 (ref.)	9.9	39.1	6.3	28.9	15.8	11.7	7.3	29.1	10.6	25.6	15.7
Parents’ social class (NS-SEC)											
I – Higher (ref.)	11.6	40.0	8.0	23.0	17.5	8.5	6.5	32.5	8.7	22.8	21.0
II – Intermediate	12.8	40.1	6.7	20.2	20.2	11.8	8.7	32.5	8.6	20.0	18.4
III – Small employers & own account	15.7	45.8	9.2	**11.3**	18.0	14.3	9.6	29.7	11.4	17.3	17.6
IV – Lower supervisory or technical	18.5	37.9	11.9	**9.1**	22.6	**18.6**	10.7	28.3	11.9	19.3	**11.3**
V – Semi-routine and routine	**23.0**	32.9	**14.2**	**15.3**	14.7	**21.4**	**18.4**	28.8	10.2	**14.1**	**7.2**
Not applicable	**27.3**	**22.6**	18.6	19.1	12.3	19.1	**19.2**	**21.1**	13.5	19.9	**7.2**
Family structure											
Living with two parents (ref.)	14.8	38.7	10.1	18.0	18.5	12.3	10.0	31.6	10.2	18.9	17.0
Living without two parents	21.0	**33.2**	11.7	20.5	13.6	**18.6**	13.8	**25.9**	8.6	21.5	**11.6**

Next Steps study (2015–16).

Estimates are adjusted marginal probabilities from a multinomial logistic regression model, weighted for non-response in 20 imputed datasets. Predictors were entered in a single model with region of residence in adolescence and ethnic group (White/Black/South Asian/Mixed & Other). Bolded estimates differ from the reference category at the *p* < 0.01 level.

*NS-SEC* national statistics socioeconomic classification. Pathways: *FF* fragile family, *SS* slow starter, *TF* traditional family, *E* educated, *W* work-oriented, *LB* left behind.

In the 1970 cohort, mother’s age at birth, parental education, parental social class, and family structure were each associated with patterns at age 26. Those who had a mother aged 30+ at birth (compared with ages 20–24) were: 1) among men, more likely to be in the slow starter group and less likely to be in the traditional family group; 2) among women, more likely to be in the educated and slow starter groups and less likely to be in the fragile family group. Those with parents who left education at ages 16 or less (compared to ages 18 or more) were, in both men and women, less likely to be in the educated group and more likely to be in the traditional family group. Those with parents whose highest occupation was a partly-skilled or unskilled job (compared with a professional job) were: 1) among men, less likely to be in the educated and work-oriented groups and more likely to be in the fragile family, slow starter, and traditional family groups; 2) among women, less likely to be in the educated and work-oriented groups and more likely to be in the fragile family and traditional family groups. Finally, those who grew up in a single parent home (compared with two-parent home) were: 1) among men, more likely to be in the educated group; 2) among women: less likely to be in the work-oriented group and more likely to be in the fragile family group.

In the Next Steps cohort, the parental characteristics were also each associated with patterns at ages 25–26. Those who had a older mother at birth were: 1) among men, more likely to be in the educated group and less likely to be in the traditional family group; 2) among women, more likely to be in the educated and slow starter groups and less likely to be in the fragile family and traditional family groups. Those whose parents did not continue education after age 16 were: 1) among men, less likely to be in the educated group and more likely to be in the left behind and traditional family groups; 2) among women, less likely to be in the educated group and more likely to be in the left behind group. Those whose parents were in a lower social class were: 1) among men, less likely to be in the educated group and more likely to be in the left behind and traditional family groups; 2) among women, less likely to be in the educated and work-oriented groups and more likely to be in the fragile family and left behind groups. Finally, those who did not grow in a two-parent home were: 1) among men, less likely to be a slow starter; 2) among women: less likely to be in the work-oriented and slow starter group and more likely to be in the fragile family group.

Comparing cohorts, there are four key differences: 1) in both sexes, being in a “slow starter” group was more common in those whose parents were in a lower social class in 1996, but not in 2015–16 (in fact, it was *less* common in those whose parents were not employed in 2015–16); 2) in both sexes, whereas parental education was not associated with being in the precarious “fragile family” group in 1996, it was negatively associated with being “left behind” in 2015–16; 3) in men, being in a work-oriented group was positively associated with parental social class in 1996, not in 2015–16; 4) in women, being in a “traditional family” group was negatively associated with parental education and social class in 1996, not in 2015–16.

## Discussion

Whereas studies have highlighted changes in trajectories within education, employment, family, and housing in more recent generations, evidence on the combination of these social roles since the late 1990s has been missing (Billari & Liefbroer, 2010, Schoon & Lyons-Amos, 2016). However, changes in employment and housing opportunities as well as patterns of family formation have likely led more recent generations to experience increasingly disaggregated pathways less well captured by past typologies (Schoon, 2015, Schoon & Lyons-Amos, 2016, 2017). This is particularly relevant for policymaking if young adults in pathways not captured in previous generations present heightened difficulties and would benefit from better-tailored policies (Finch, 2008, Crisp & Powell, 2017). Comparing role combinations patterns at ages 25–26 in 1996 and 2015–16 among those who grew up in England, the findings suggest that trends in education, employment, family, and housing over the past twenty years have been associated with substantial changes for young adults.

The re-analysis of experiences in the 1970 cohort supports that the transitions of British men and women in the 1990’s likely occurred through one of five clear pathways. For the more recent cohort born in 1989–90, LCA led us to identify a different set of patterns, which were not fully representative of previous generations’ experiences. The size of the work-oriented home-owner group decreased by 50% between 1996 and 2015–16. The traditional family group also characterized a smaller proportion of the cohort, and was no longer associated with home ownership in men in 2015–16. In contrast, the slow starter group grew in size by 20% in men and 50% in women. Finally, a new “left behind” group emerged, representing one in six men and one in nine women in 2015–16.

A cross-cutting decrease was found in the size of positive pathways (i.e., “work-oriented” in both genders, “traditional family” in women) and increase in the size of less desirable pathways in terms of financial independence and relative security (i.e., “slow starter” and “left behind” in both genders, “fragile family” in women). Three findings support the idea of increasing de-standardization and precarization in youth transitions: 1) milestones that changed in prevalence – i.e., home ownership, marriage in men, and higher education in women – did not delineate pathways as well in 2015–16 as they did in 1996; 2) changes in men’s and women’s milestones over time led to more pronounced gender differences in role combinations in 2015–16 compared with 1996; 3) a new precarious pattern of being “left behind”, i.e., young adults who are less likely to attain post-secondary qualifications and full-time employment and are excluded from home ownership and the “relationship market”, has now become a relatively common reality at this age. In keeping with this shift in transition patterns, new values may have become attached to the transition milestones that have become less attainable over time, and influence in new ways identity fulfillment and psychological well-being.

Beyond changes in the size and nature of pathways, inequalities by family background were generally similar across cohorts. Supporting previous studies using the 1970 British Cohort study, mother’s age at birth, parents’ education and social class, and family structure in adolescence each predicted young adults’ capacity to enter education- and work-oriented pathways and postpone family-oriented pathways by age 26 in 1996 (Ross et al., 2009; Schoon et al., 2012). Supporting the persistence of inequalities, we found in the 1989–90 cohort that those in the “educated” group had a more advantaged family background compared with those in the “left behind” and “fragile family” groups.

Three key changes were found in the social distribution of role combinations at ages 25–26 between 1996 and 2015–16. The first concerns the “left behind” group, which was strongly associated with parental characteristics in the more recent cohort. Young men and women in this group were likely to have grown up in separated families where parents had few qualifications and routine jobs. In comparison, among young adults in fragile families in 1996, the magnitude of the influence of parents’ education was relatively small (Schoon et al., 2012). This suggests that the risk of entering a precarious pathway may have disproportionally increased among less privileged groups, suggesting an increased polarization of young adult transitions over time. Those in the emerging “left behind” pathway may experience more difficulties and new needs in making the transition to new social roles compared with what could have been gleaned from previous generations.

The second change across cohorts concerns the “slow starters” group, which was no longer associated with social class of origin in 2015–16 whereas it was more common among men in less privileged families (i.e., working in “semi-skilled and unskilled” jobs) in 1996. Given the increase in the size of this group in both genders across cohorts, the “slow starters” group may capture an increasingly heterogeneous group that benefits less from parental resources and has to delay transitions towards independent living in response to labor and housing market pressures (Côté & Bynner, 2008; Côté, 2014). This finding contrasts from previous studies that found that “slow starters” in 1996 were *less* likely in those from less privileged families (Schoon et al., 2012). Key differences in the models here included not adjusting for three characteristics at ages 16: school motivation, expectations towards education, and exam scores. It could be that, adjusting for these mediators, the remaining (direct) effect of social class of origin was negative: that is, whereas men in the 1970 cohort performed better in education if they were from a more privileged family (which would lead them towards the “educated” or “work-oriented” pathways), this privilege could also lead them to actively delay their transition out of the parental home (i.e., there is a residual effect of social class predicting being “slow starter” once the additional variables are included in the model).

Finally, there were no longer differences by parental indicators for those in a “work-oriented” group among men and those in a “traditional family” group among women in 2015–16, whereas these patterns were associated with social class of origin in 1996. Given that these patterns are defined in part by home ownership, parental resources were expected to be associated with young adults’ capacity to purchase their first property, which was however not the case for men. The findings may suggest that “work oriented” and “traditional family” patterns are changing their meaning for current cohorts of young people, becoming more “desirable” among both the relative privileged and less privileged.

With fewer young people in secure employment, cohabiting with a partner, and able to afford a house, policymakers must address the realities of new generations planning to start independent living and family formation, and struggling in the labor market. Policy examples for employment include improving the match between the provision of education and local labor market opportunities, re-distributing funds to deprived areas away from metropolitan cities, and reducing inequalities—beyond access to higher education—in drop out rates, degrees pursued, and the risk of working in a job for which one is overqualified (UKSMC, 2020). This also includes regulating against zero-hour jobs and temporary, precarious employment to help young people have a predictable and viable income. Policy examples for families include the expansion of eligibility for subsidized childcare and the better marketing of services to marginalized communities, as well as investments in the creation of affordable housing (UKSMC, 2019).

This study builds on the methodological qualities of the 1970 British Cohort Study and the Next Steps study to produce representative evidence on role combinations at the ages of 25–26 among those who grew up in England. Results for England may not match young adult experiences in other Western countries. Regarding limitations, pathways were derived using a single time point and did not consider the timing and sequence of transitions despite their importance in capturing the complexity of young people’s experiences (Schoon & Lyons-Amos, 2016, 2017). Readers should also be careful in attaching too much meaning to a latent class or the label assigned to it (MacMillan & Eliason, 2003). These models provide only a summary of the ways in which role configurations may occur in society.

## Conclusion

For those born in more recent years, the mid-20’s are shaped by the continued rise of higher education participation, decreases in full-time employment and home ownership, and delays in moving out of the parental home and in with partners, obscuring our understanding of recent pathways to adulthood. The findings highlight important changes in role combinations at ages 25–26 across two cohorts who grew up in England 20 years apart. The transition to adulthood is more complex and risk-laden, with increasing numbers of “slow starters” delaying independent living, the emergence of a new group of young adults “left behind” in labor and relationship markets, and increased inequalities in avoiding the more precarious role configurations. The evidence built on earlier generations may only partially apply to more recent generations, challenging our understanding of the extent to which the transition to adulthood may determine identity development and psychological well-being in more recent years.

## Supplementary information

Supplementary Document
