# Supplementary material for: Changes in Patterns of Social Role Combinations at Ages 25–26 among Those Growing Up in England between 1996 and 2015–16: Evidence from the 1970 British Cohort and Next Steps Studies
Source: J Youth Adolesc. 2021 Jul 16;50(10):2052–66. doi: 10.1007/s10964-021-01477-1 (PMC8416831; doi:10.1007/s10964-021-01477-1)
Supplement: Supplementary file 1 — Supplementary Document [file 10964_2021_1477_MOESM1_ESM.docx]

**SUPPLEMENTARY DOCUMENT**

Changes in Patterns of Social Role Combinations at Ages 25-26 among Those Growing Up in England between 1996 and 2015-16. Evidence from the 1970 British Cohort and Next Steps Studies.

**1. Sample characteristics, frequencies and missing cases**

**2. Distribution of predictors in the 1970 British Cohort study**

**2. Distribution of predictors in the Next Steps study**

**3. Comparing class solutions in the 1970 British Cohort study**

**4. Comparing class solutions in the Next Steps study**

**Supplementary Table 1. Sample characteristics in the 1970 British Cohort (1996) and Next Steps (2015-16) studies.**

|  | **1970 BCS**  ***N* = 7,112** | | | | **Next Steps**  ***N* = 7,707** | | | |
| --- | --- | --- | --- | --- | --- | --- | --- | --- |
| **Variable** | **Men**  ***N* = 3,191** | | **Women**  ***N* = 3,921** | | **Men**  ***N* = 3,246** | | **Women**  ***N* = 4,281** | |
|  | **N** | **%** | **N** | **%** | **N** | **%** | **N** | **%** |
| **Education (NVQ)**  No qualifications  NVQ 1: CSE  NVQ 2: 0-level  NVQ 3: A-level  NVQ 4: Higher qual  NVQ 5: Degree  *Missing* | 328  206  840  462  891  152  312 | 10.3  6.5  26.3  14.5  27.9  4.8  9.8 | 439  324  1,177  443  1,038  198  302 | 11.2  8.3  30.0  11.3  26.5  5.1  7.7 | 256  387  794  632  805  550  2 | 7.5  11.3  23.2  18.5  23.5  16.1  0.1 | 287  349  884  874  1,185  695  7 | 6.7  8.2  20.7  20.4  27.7  16.2  0.2 |
| **Housing tenure**  Main owner  Rent  Rent-free & other  *Missing* | 1,238  889  1,014  50 | 38.8  27.9  31.8  1.6 | 1,823  1,158  890  50 | 46.5  29.5  22.7  1.3 | 705  1,259  1,446  16 | 20.6  36.8  42.2  0.5 | 969  1,818  1,468  26 | 22.6  42.5  34.3  0.6 |
| **Living with parents**  Living with parents  Living without parents  *Missing* | 870  2,321  0 | 27.3  72.7 | 748  3,173  0 | 19.1  80.9 | 1,303  2,121  2 | 38.0  61.9  0.1 | 1,313  2,965  3 | 30.7  69.3  0.1 |
| **Economic activity**  FT employed  PT employed  Unemployed  FT student  At-home  Other  *Missing* | 2,665  79  222  93  11  93  28 | 83.5  2.5  7.0  2.9  0.3  2.9  0.9 | 2,515  490  103  81  545  134  53 | 64.1  12.5  2.6  2.1  13.9  3.4  1.4 | 2,641  250  256  164  14  78  23 | 77.1  7.3  7.5  4.8  0.4  2.3  0.7 | 2,727  620  246  228  351  79  30 | 63.7  14.5  5.8  5.3  8.2  1.9  0.7 |
| **Relationship status**  Single  In couple  Married  Div./Sep./Widowed  *Missing* | 1,573  777  752  59  30 | 49.3  24.4  23.6  1.9  0.9 | 1,353  996  1,359  191  22 | 34.5  25.4  34.7  4.9  0.6 | 2,321  786  303  10  6 | 67.8  22.9  8.8  0.3  0.2 | 2,457  1,165  611  43  5 | 57.4  27.2  14.3  1.0  0.1 |
| **Living with children**  No children  One child  Two or more children  *Missing* | 2,674  347  170  0 | 83.8  10.9  5.3 | 2,752  656  513  0 | 70.2  16.7  13.1 | 3,036  239  151  0 | 88.6  7.0  4.4  0 | 3,207  587  487  0 | 74.9  13.7  11.4  0 |

NVQ = National Vocational Qualifications. FT = Full-time. PT = Part-time. Div. = Divorced. Sep. = Separated

**Supplementary Table 2. Distribution of regressors in the 1970 British cohort.**

|  | **1970 BCS**  **N = 7,112** | | | | | |
| --- | --- | --- | --- | --- | --- | --- |
| **Variable** | **M**  ***N* = 3,191** | | | **F**  ***N* = 3,921** | | |
|  | **N** | **Raw%** | **W%** | **N** | **Raw%** | **W%** |
| **Mother’s age at birth**  Less than 20  20-24  25-29  30 or more  *Missing* | 279  1,137  1,021  731  23 | 8.7  35.6  32.0  22.9  0.7 | 10.0  35.7  30.5  23.7 | 281  1,421  1,297  911  11 | 7.2  36.2  33.1  23.2  0.3 | 8.1  36.3  31.7  23.9 |
| **Parental education**  Left FT educ. at ages ≤ 16  Left FT educ. at ages 17-18  Left FT educ. at ages ≥ 19  *Missing* | 2,152  567  460  12 | 67.4  17.8  14.4  0.4 | 71.1  16.3  12.6 | 2,655  712  545  9 | 67.7  18.2  13.9  0.2 | 70.5  16.9  12.6 |
| **Parental social class**  RGSC I  RGSC II  RGSC III  RGSC IV  RGSC V  Not applicable  *Missing* | 332  1,019  1,604  163  39  34  0 | 10.4  31.9  50.3  5.1  1.2  1.1  0 | 8.8  29.7  51.6  6.4  1.8  1.6 | 385  1,312  1,938  211  45  30  0 | 9.8  33.5  49.4  5.4  1.2  0.8 | 8.7  31.6  50.8  6.3  1.5  1.0 |
| **Family structure**  Living with both parents  Not living with both parents  *Missing* | 2,466  595  130 | 77.3  18.7  4.1 | 79.2  20.8 | 3,065  716  140 | 78.2  18.3  3.6 | 80.0  20.0 |
| **Region in adolescence**  North  Yorkshire & Humberlands  East Midlands  East Anglia  South East  South West  West Midlands  North West  *Missing* | 239  332  281  160  1,084  273  385  437  0 | 7.5  10.4  8.8  5.0  34.0  8.6  12.1  13.7  0 | 7.8  10.6  8.8  5.0  33.6  8.1  12.0  14.0 | 284  446  295  191  1,295  406  438  566  0 | 7.2  11.4  7.5  4.9  33.0  10.4  11.2  14.4 | 7.4  11.6  7.4  4.8  32.7  10,0  11.2  14.7 |
|  |  |  |  |  |  |  |
| **Ethnic group**  British/Irish  Other  *Missing* | 2,970  152  69 | 93.1  4.8  2.2 | 94.1  5.9 | 3,675  178  68 | 93.7  4.5  1.7 | 94.6  5.4 |
|  |  |  |  |  |  |  |

Mother’s age at birth and ethnic group were measured at age 0 whereas parental education, parental social class, family structure, and region in adolescence were measured at age 16, and at ages 0-10 among those with missing data at ages 16.

W% = Proportion weighted with the author-created non-response weight based on birth variables in the 1970 cohort.

**Supplementary Table 3. Distribution of regressors in the Next Steps cohort.**

|  | **Next Steps**  **N = 7,707** | | | | | |
| --- | --- | --- | --- | --- | --- | --- |
| **Variable** | **M**  ***N* = 3,246** | | | **F**  ***N* = 4,281** | | |
|  | **N** | **Raw%** | **W%** | **N** | **Raw%** | **W%** |
| **Mother’s age at birth**  Less than 20  20-24  25-29  30 or more  *Missing* | 202  784  1,150  1,098  192 | 5.9  22.9  33.6  32.1  5.6 | 7.8  25.5  35.3  31.4 | 275  997  1,379  1,401  229 | 6.4  23.3  32.2  32.7  5.4 | 8.4  25.9  33.3  32.5 |
| **Parental education**  Left FT educ. at ages ≤ 16  Left FT educ. at ages 17-18  Left FT educ. at ages ≥ 19  *Missing* | 1,284  808  1,232  102 | 37.5  23.6  36.0  3.0 | 38.3  24.3  37.4 | 1,645  1,008  1,472  156 | 38.4  23.6  34.4  3.6 | 38.8  25.3  35.9 |
| **Parental social class**  NS-SEC I  NS-SEC II  NS-SEC III  NS-SEC IV  NS-SEC V  Not applicable  *Missing* | 1,489  350  363  306  647  157  114 | 43.5  10.2  10.6  8.9  18.9  4.6  3.3 | 50.2  11.0  10.0  8.5  17.4  2.9 | 1,793  477  417  361  801  276  156 | 41.9  11.1  9.7  8.4  18.7  6.5  3.6 | 48.8  12.4  9.5  8.8  17.4  3.1 |
| **Family structure**  Living with both parents  Not living with both parents  *Missing* | 2,669  683  74 | 77.9  19.9  2.2 | 72.2  27.8 | 3,209  952  120 | 75.0  22.2  2.8 | 70.7  29.3 |
| **Region in adolescence**  North East  North West  Yorkshire & Humberlands  East Midlands  West Midlands  East of England  London  South East  South West  *Missing* | 145  479  301  289  371  352  480  454  257  298 | 4.2  14.0  8.8  8.4  10.8  10.3  14.0  13.3  7.5  8.7 | 5.8  15.8  9.2  9.8  11.7  10.3  12.7  15.0  9.7 | 191  518  420  330  447  410  693  585  308  379 | 4.5  12.1  9.8  7.7  10.4  9.6  16.2  13.7  7.2  8.9 | 6.1  13.6  11.1  9.4  11.1  10.4  13.5  15.4  9.6 |
|  |  |  |  |  |  |  |
| **Ethnic group**  White  Black  South Asian  Mixed / Other  *Missing* | 2,370  177  593  228  58 | 69.2  5.2  17.3  6.7  1.7 | 85.5  2.9  6.3  5.2 | 2,885  261  724  319  92 | 67.4  6.1  16.9  7.5  2.2 | 85.3  3.5  5.8  5.4 |
|  |  |  |  |  |  |  |

All variables were measured at the ages 13-14 baseline.

W% = Proportion weighted with the non-response weight created by the Next Steps team in the Next Steps cohort.

**Supplementary Table 3. Fit of solutions for patterns at age 26 in those who grew up in england in the 1970 British Cohort Study (1996).**

|  | Males (*n* = 3,191) | | | | | Females (*n* = 3,921) | | | | |
| --- | --- | --- | --- | --- | --- | --- | --- | --- | --- | --- |
| Classes | BIC | SA BIC | Entropy | VLMR | %  smallest  group | BIC | SA BIC | Entropy | VLMR | %  smallest  group |
| 2 | 31816 | 31698 | .880 | < .001 | 32.5 | 44976 | 44859 | .939 | < .001 | 19.6 |
| 3 | 31126 | 30948 | .828 | < .001 | 27.8 | 42494 | 42316 | .920 | < .001 | 19.6 |
| 4 | **30872** | 30634 | .860 | **.538** | 16.5 | 42005 | 41767 | .875 | **.746** | 16.5 |
| 5 | 30884 | **30585** | .868 | .736 | **3.1** | **41870** | 41572 | .850 | .773 | 12.2 |
| 6 | --- | --- | --- | --- | --- | 41900 | **41541** | .862 | .809 | **1.5** |
| 7 | --- | --- | --- | --- | --- | --- | --- | --- | --- | --- |

Estimates come from latent class analyses of educational attainment, housing tenure, cohabitation with parents, economic activity, relationship status, and parenthood in men and women, weighted for non-response using FIML. The “% smallest group” value is weighted and based on the prevalence when participants are classified according to their most likely class.

SA BIC = Sample-size-adjusted Bayesian Information Criteria. VLMR = Vuong-Lo-Mendell-Rubin test.

**Supplementary Table 4. Fit of solutions for patterns at ages 25-26 in the Next Steps Study (2015-16).**

|  | Males (*n* = 3,426) | | | | | Females (*n* = 4,281) | | | | |
| --- | --- | --- | --- | --- | --- | --- | --- | --- | --- | --- |
| Classes | BIC | SA BIC | Entropy | VLMR | % smallest group | BIC | SA BIC | Entropy | VLMR | % smallest group |
| 2 | 37256 | 37138 | 0.84 | < .001 | 32.6 | 52159 | 52042 | 0.88 | < .001 | 31.2 |
| 3 | 36819 | 36641 | 0.79 | < .001 | 14.6 | 50543 | 50365 | 0.80 | < .001 | 30.0 |
| 4 | 36703 | 36465 | 0.73 | **0.76** | 13.5 | 50309 | 50071 | 0.79 | **0.07** | 9.9 |
| 5 | **36594** | 36296 | 0.72 | 0.89 | 10.5 | 50157 | 49858 | 0.78 | 0.29 | 8.7 |
| 6 | 36641 | **36282** | 0.74 | 0.77 | **2.8** | 50119 | 49760 | 0.75 | 0.78 | 9.6 |
| 7 | --- | --- | --- | --- | --- | **50091** | 49672 | 0.77 | 0.85 | **2.4** |
| 8 | --- | --- | --- | --- | --- | 50132 | **49652** | 0.78 | 0.76 | 2.4 |

Estimates come from latent class analyses of educational attainment, housing tenure, cohabitation with parents, economic activity, relationship status, and parenthood in men and women, weighted for non-response using FIML. The “% smallest group” value is weighted and based on the prevalence when participants are classified according to their most likely class.

SA BIC = Sample-size-adjusted Bayesian Information Criteria. VLMR = Vuong-Lo-Mendell-Rubin test.
